# Complete Genome Sequences of Mycobacteriophages Clautastrophe, Kingsolomon, Krypton555, and Nicholas

**DOI:** 10.1128/genomeA.01129-17

**Published:** 2017-11-09

**Authors:** Hui-Min Chung, Tom D’Elia, Joseph F. Ross, Samuel M. Alvarado, Molly-Catherine Brantley, Lydia P. Bricker, Courtney R. Butler, Carson Crist, Julia M. Dane, Brett W. Farran, Sierra Hobbs, Michelle Lapak, Conner Lovell, Nicholas Ludergnani, Allison McMullen, Sohail A. Mirza, Noah Thrift, Donald P. Vaughan, Grace Worley, Amara Ejikemeuwa, May Zaw, Claude F. Albritton, Sarah C. Bertrand, Shanzay S. Chaudhry, Vzair A. Cheema, Camilla Do, Michael L. Do, Huyen M. Duong, Dalia H. El-Desoky, Kelsey M. Green, Rhea N. Lee, Lauren A. Thornton, James M. Vu, Mah Noor Zahra, Steven G. Cresawn, Ty H. Stoner, Rebecca A. Garlena, Deborah Jacobs-Sera, Welkin H. Pope, Daniel A. Russell, Graham F. Hatfull

**Affiliations:** a Department of Biology, University of West Florida, Pensacola, Florida, USA; b Department of Biology, Indian River State College, Fort Pierce, Florida, USA; c Department of Biology, Xavier University of Louisiana, New Orleans, Louisiana, USA; d Department of Biology, James Madison University, Harrisonburg, Virginia, USA; e Department of Biological Sciences, University of Pittsburgh, Pittsburgh, Pennsylvania, USA

## Abstract

We report here the complete genome sequences of four subcluster L3 mycobacteriophages newly isolated from soil samples, using *Mycobacterium smegmatis* mc^2^155 as the host. Comparative genomic analyses with four previously described subcluster L3 phages reveal strong nucleotide similarity and gene conservation, with several large insertions/deletions near their right genome ends.

## GENOME ANNOUNCEMENT

Mycobacteriophages are viruses infecting *Mycobacterium*, a bacterial genus commonly found in soil and water (1). A collection of over 1,300 sequenced genomes of phages infecting *Mycobacterium smegmatis* mc^2^155 has accumulated through the work of students in the Science Education Alliance Phage Hunters Advancing Genomics and Evolutionary Science (SEA-PHAGES) program, revealing mycobacteriophages to be highly diverse and to have complex relationships (2). These phages can be grouped by their relatedness into 25 clusters and 6 singletons (those with no close relatives) (3). We report here the genome sequences of mycobacteriophages Kingsolomon and Nicholas, isolated in Gulf Breeze, FL, and phages Clautastrophe and Krypton555, isolated in greater New Orleans, LA.

Phages Clautastrophe, Kingsolomon, Krypton555, and Nicholas were isolated either by direct plating or enrichment of soil samples using *M. smegmatis* mc^2^155 as the host, followed by plaque purification and amplification; electron microscopy showed that Kingsolomon and Nicholas are members of the family *Siphoviridae*. Following the isolation of double-stranded DNA, genomes were sequenced using the Illumina MiSeq platform, and 150-base single-end reads were assembled using Newbler and Consed (4); coverage exceeded 1,800-fold for all genomes, and all have defined genome termini with 10-base complementary 3′ single-strand DNA extensions (right end, 5′-TCGATCAGCC). Genome annotations were performed using DNA Master (http://cobamide2.bio.pitt.edu/computer.htm), Phamerator (5), Glimmer (6), GeneMark (7), Aragorn (8), and tRNAscan-SE (9), and functional assignments were predicted using BLASTP (10) and HHpred (11). Repetitive-sequence motifs were identified using the MEME Suite software (12) and Geneious 10.1.3 (13).

All four genomes are closely related and similar to other subcluster L3 phages; all have similar GC% contents (59.1 to 59.3%). Genome lengths (72,055 bp, 76,075 bp, 70,413 bp, and 69,537 bp), numbers of protein-coding genes (122, 130, 112, and 110), and numbers of tRNA genes (10, 11, 10, and 10) were assigned for Clautastrophe, Krypton555, Nicholas, and Kingsolomon, respectively. The Nicholas and Kingsolomon genomes are almost identical, but Kingsolomon has an 876-bp deletion relative to Nicholas near its right genome end; Clautastrophe and Krypton555 differ from other L3 genomes by at least 15 separate insertions/deletions. All four genomes have features common to temperate phages, including genes encoding putative repressors (e.g., Krypton555 *38*) and integrases (e.g., Krypton555 *36*); *attP* sites have not been identified. Functional assignments are predicted for up to 40% of the gene products.

Clautastrophe, Kingsolomon, and Nicholas have deletions near their right genome ends relative to Krypton555 and other subcluster L3 phages, missing approximately 4.3 kbp, 6.1 kbp, and 6.1 kbp, respectively. This region is characterized by multiple copies of different repeat sequences. For example, Krypton555 contains four identical copies of both 5′-TACACGCCGTGACCG and 5′-CGGAGCTGCTTGGTT, many of which are missing from the other three genomes. The roles of these repeat sequences are unknown, but they could be involved in the regulation of early lytic gene expression, which is predicted to initiate near the right genome end. Although most of the genes deleted from Clautastrophe, Kingsolomon, and Nicholas are of unknown function, one deleted gene present in Krypton555 and the L3 phages Lumos, Snenia, and Whirlwind codes for an MCM2-like AAATPase, which is presumably not essential for lytic growth in these phages.

### Accession number(s).

The nucleotide sequence accession numbers are MF140405 for Clautastrophe, MF140413 for Kingsolomon, MF140414 for Krypton555, and MF140421 for Nicholas.
